# A Mobile Patient-Facing App for Tracking Patient-Reported Outcomes in Head and Neck Cancer Survivors: Single-Arm Feasibility Study

**DOI:** 10.2196/24667

**Published:** 2021-03-19

**Authors:** Sewit Teckie, Jeffrey Solomon, Karthik Kadapa, Keisy Sanchez, David Orner, Dennis Kraus, Dev P Kamdar, Lucio Pereira, Douglas Frank, Michael Diefenbach

**Affiliations:** 1 Academic Department of Radiation Medicine Donald and Barbara Zucker School of Medicine at Hofstra/Northwell New York, NY United States; 2 Department of Radiation Medicine Northwell Health Cancer Institute Lake Success, NY United States; 3 Center for Health Innovations and Outcomes Research Department of Medicine Northwell Health Manhasset, NY United States; 4 Center for Research Informatics & Innovation Feinstein Institutes for Medical Research Northwell Health Manhasset, NY United States; 5 Department of Otolaryngology Donald and Barbara Zucker School of Medicine at Hofstra/Northwell Hempstead, NY United States; 6 Department of Otolaryngology Northwell Health Cancer Institute Lake Success, NY United States

**Keywords:** mHealth, ePROs, head and neck cancer, mobile phone

## Abstract

**Background:**

Patients with head and neck cancer (HNC) frequently experience disease-related symptoms and treatment adverse effects that impact their overall quality of life. Cancer-specific mobile health apps for patient-related outcomes allow patients to communicate with their clinicians and proactively track their symptoms, which have been shown to improve clinical management and disease outcomes.

**Objective:**

The purpose of this study was to evaluate the feasibility of LogPAL, a novel iPhone-based mobile health app designed to help HNC survivors track and manage their posttreatment symptoms.

**Methods:**

Patients who completed curative treatment for HNC in the preceding 24 months were recruited from 2 clinical sites within a single institution. Upon enrollment, participants completed a brief sociodemographic survey, downloaded the app onto their iPhone devices, and were asked to complete a series of biweekly questionnaires (based on the Patient-Reported Outcomes version of the Common Terminology Criteria for Adverse Events) via the app for an 8-week study period. The primary feasibility endpoints included retention (retaining >80% of the enrolled participants for the duration of the study period), adherence (>50% of the participants completing 100% of the questionnaires over the study period), and usability (a mean system usability scale [SUS] score >68). Additional postintervention questions were collected to assess perceived usefulness, acceptance, and overall satisfaction.

**Results:**

Between January and October 2019, 38 participants were enrolled in the study. Three participants dropped out, and 3 were classified as nonusers. The remaining 32 (87%) were eligible for analysis. Their mean age was 57.8 (SD 12.3) years (range 24-77 years, 81% [26/32] male). Overall, 375 of 512 (73.2%) questionnaires were completed, with 17 (53%) of the 32 participants adherent. Participant-reported usability was acceptable; the mean SUS score was 71.9 (95% CI 64.3-79.5) with high satisfaction of LogPAL usefulness and likelihood to recommend to other cancer survivors.

**Conclusions:**

This single-arm prospective pilot study showed that LogPAL is a feasible, regularly used, accepted app for HNC survivors, justifying a full-scale pilot. Based on the findings from this study, future iterations will aim to improve usability and test intervention efficacy.

## Introduction

### Background

The demographics of head and neck cancer (HNC) are changing [1-4], with an increase in the incidence of HNC in younger patients without significant smoking or alcohol use history. HNC survival rates are increasing as a result of these changing demographics and more effective multidisciplinary treatment options [5-8]. Despite these advances in disease outcomes, HNC survivors often experience significant toxicities and unique functional impairments such as dysphagia, mucositis, xerostomia, and dysphonia, which are distinctive from those reported in other cancer survivors [9-11]. These can lead to debilitating and lifelong consequences due to adverse effects during and after treatment, which impair the quality of life. Nevertheless, patients commonly underreport symptoms or delay reporting of symptoms, which, in turn, lead to delay in the best clinical management [12,13]. Mobile health (mHealth) interventions such as smartphone apps have been advocated as promising strategies in patient self-management [14]. These tools have the potential to increase accessibility of patient’s health information, provide real-time reporting of the concerning symptoms to providers, and improve overall patient satisfaction via proactive self-management care [15-19]. The proliferation of mHealth apps, with over 325,000 mHealth apps developed [20], is changing how patients interact with the health care system. Yet, there remains a dearth in knowledge regarding the feasibility of using mHealth electronic patient-reported outcomes (ePROs) to assess and address the vulnerable population of HNC survivors. To date, only few studies have evaluated a smartphone-based self-management system during HNC treatment [16,21]. To fill this knowledge gap, our study aimed to evaluate the feasibility of LogPAL, a novel patient-facing mHealth app specific for HNC survivors.

### Objectives of This Study

The primary objectives of this study were to (1) assess the feasibility of LogPAL and (2) explore the perceived usefulness, acceptance, and overall satisfaction through validated questionnaires and participant feedback. We hypothesized that there would be an >80% retention rate (proportion of enrolled participants who completed at least one questionnaire during the study period), >50% adherence rate (proportion of participants who will complete 100% of scheduled questionnaires), and a mean system usability scale (SUS) score >68. Secondary exploratory objectives reviewed additional engagement metrics and preliminary associations between outcome measures and participant characteristics (covariates). As there is no consensus on how best to evaluate mHealth pilot studies, criteria selection and determination were based on insights from the one of the co-principal investigators (MAD) who is experienced in evaluating web-based and app-based health tools for patients with cancer.

## Methods

### Recruitment and Enrollment

All HNC survivors were screened for eligibility from 2 Northwell Health otolaryngology surgical oncology clinics. Potential participants were identified through a review of electronic medical records to determine if the following inclusion criteria were met prior to their upcoming in-clinic appointment: HNC survivors ≥18 years old who completed all curative treatments for primary HNC (lip/oral cavity, pharynx, larynx, salivary gland, paranasal sinus) within the preceding 24 months, who had no serious self-reported cognitive impairment, who were able to read and speak English fluently, and who had access to a smartphone that operates the iPhone operating system software (iPhone/iPad). Eligible participants were contacted by phone to discuss meetings about enrollment at the clinic during an upcoming appointment. Upon meeting, additional details about the study were reviewed and participants were informed that participation was voluntary. If informed consent was provided, participants underwent a training session with a study team member on how to use the app, with additional time for any questions prior to the first log-in.

### Study Design

A nonrandomized, prospective, single-arm pilot study was carried out from January to October 2019. The study period consisted of an 8-week intervention during which participants were instructed to answer and complete ePROs twice weekly for 16 sessions. The frequency and duration of the ePROs were determined by a team of HNC specialists to reflect clinically relevant timepoints that encouraged self-monitoring, but did not compromise HNC patient safety during their recovery. This study was reviewed and approved by the Institutional Review Board of Northwell Health.

### LogPAL App

#### LogPAL Development

The LogPAL app was developed using an iterative user-centered design approach [22,23], which systematically engaged end users to identify requirements and app core functionality to enhance the effectiveness, efficiency, and usability [24,25]. The goal was to develop a patient-facing mHealth app that could provide HNC survivors with a tool to better manage and track their symptoms by self-reporting any adverse effects and offer immediate informational resources to help develop and practice health self-management skills.

#### LogPAL Overview

**Figure 1:**
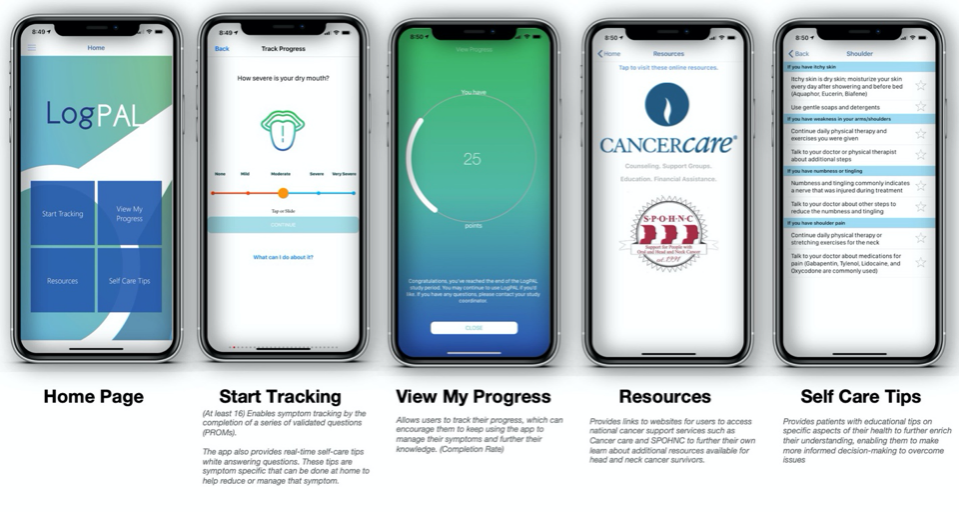
Snapshots and overview of the LogPAL app features.

For this pilot study, a version of LogPAL, currently available in the Apple App Store, that consisted of the following 4 core features was used: (1) Start Tracking, (2) View My Progress, (3) Self Care Tips, and (4) Resources (Figure 1). Users were not specifically required to access other features other than *Start Tracking* during the intervention period.

### Structure of the PROs

**Figure 2:**
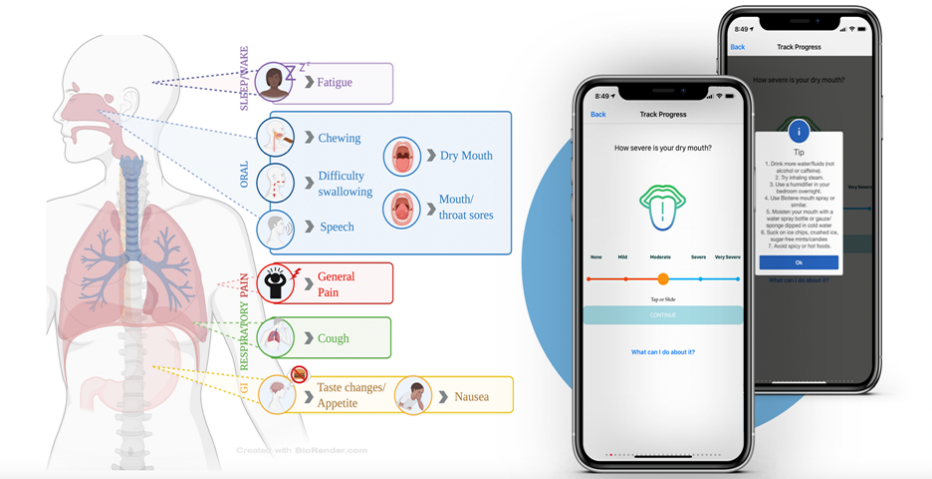
Examples of patient-reported outcome questions and illustrations of the relevant adverse effects. (Figure created on BioRender).

A preliminary list of PROs was selected from the existing validated National Cancer Institute’s Patient-Reported Outcomes version of the Common Terminology Criteria for Adverse Events library [26]. Individual items were selected by the study team for their relevance to symptoms and complications experienced by patients with HNC. To allow for iterative refinement, 2-week usability testing sessions were conducted using a purposive cohort of patients with HNC. At the end of the 2-week period, participants engaged in focus group interviews, which used the think-aloud method to elicit feedback regarding the perceived understanding of PRO questions and overall experience using the app. PROs were rated on a 5-tier scale ranging from “none” to “very severe.” Questions were categorized into 10 common disease-specific symptoms, which included difficulty swallowing and chewing, dry mouth, loss of appetite, changes in taste, impaired speech, sores/pain in the mouth, overall pain, cough, nausea, and fatigue (Figure 2). Through this careful selection process, 2 versions were created. A weekly questionnaire (consisting of a series of 26 symptom-based questions) and a monthly questionnaire (weekly questionnaire with an additional 16 questions) were asked at the end of every month.

### Data Collection

Questionnaires and app-generated data analytics were used to record participant sociodemographic characteristics at baseline, to measure engagement throughout the study period, and to perform postintervention analysis. Questionnaire data were entered into the Research Electronic Data Capture (REDCap) software and encrypted and stored in a secure server, which was selected and approved by the Office of the Chief Information Officer. REDCap’s web-based app uses secure 2-factor web authentication, data logging, and encryption that ensures the security and confidentiality of private information for obtaining informed consent [27].

### Measures

#### Patient Demographics

Participants completed an 18-item survey at enrollment. The survey included demographic characteristics about the participant’s sex, racial and ethnic background, highest level of education, and marital status. Health-related information about the treatment type received, year of diagnosis, number of years since last treatment, smoking history, self-reported physical health and physical activity relative to peers, comorbidities, and clinic site of enrollment were also obtained. Self-reported physical health and physical activity relative to peers were determined using a 5-point Likert scale (excellent, very good, good, fair, and poor) and a 3-point Likert scale (more, about the same, less), respectively.

#### Follow-up Survey

After 8 weeks of using the app, participants were invited to complete a postintervention survey via email. The survey comprised of (1) the validated SUS, a standardized questionnaire commonly used to assess participants’ perceptions of usability of an electronic system or device [28,29]; (2) a 5-point Likert response scale (1=strongly disagree to 5=strongly agree) acceptability questions; and (3) open-ended questions to prompt ideas for app improvement.

#### Outcome Measures

For this feasibility study, a priori criteria were defined as ≥80% retention rate (defined as the number of participants who enrolled and completed at least one questionnaire during the study period), ≥50% adherence rate (defined as the percentage of participants who completed all scheduled sessions), and mean SUS>68. This criterion was based on prior studies that assessed feasibility in applied intervention research [30-32] and self-management apps among cancer survivors [33]. To assess implementation outcomes such as recruitment and retention, rates were tabulated based on data collected from the research team tracking logs and summarized using a CONSORT diagram. Secondary exploratory outcomes tracked additional engagement metrics such as number of log-ins and frequency of participant interactions with other features [34]. Insights on different types of engagement indicators could provide opportunities for designing more engaging and clinically effective mHealth interventions [35].

### Statistical Analysis

For this feasibility study, 50 participants were sought to review the app. This estimate was drawn on prior experiences accruing participants [36]. Taking into consideration a retention rate of 80% of recruited patients, it is estimated that 41 patients will remain in the study. All statistical analyses were conducted using Prism 8.4.0 (GraphPad Software) and SPSS version 25.0 (IBM Corp). Descriptive statistics was utilized to summarize participant characteristics and rates of engagement. Categorical variables were reported as frequencies (n) and percentages (%); continuous variables were reported as mean (SD) or median (IQR), that is, 25th-75th percentile, as needed. Nonparametric Mann-Whitney *U* test for 2 independent groups or the Kruskal-Wallis test for more than 2 groups were used for exploratory analyses of adherence scores (out of 16). Qualitative responses to open-ended questions were categorized using a thematic analysis, and relevant quotations were included to illustrate those themes. Results with *P* values less than .05 were considered to be statistically significant.

## Results

### Recruitment and Enrollment

**Figure 3:**
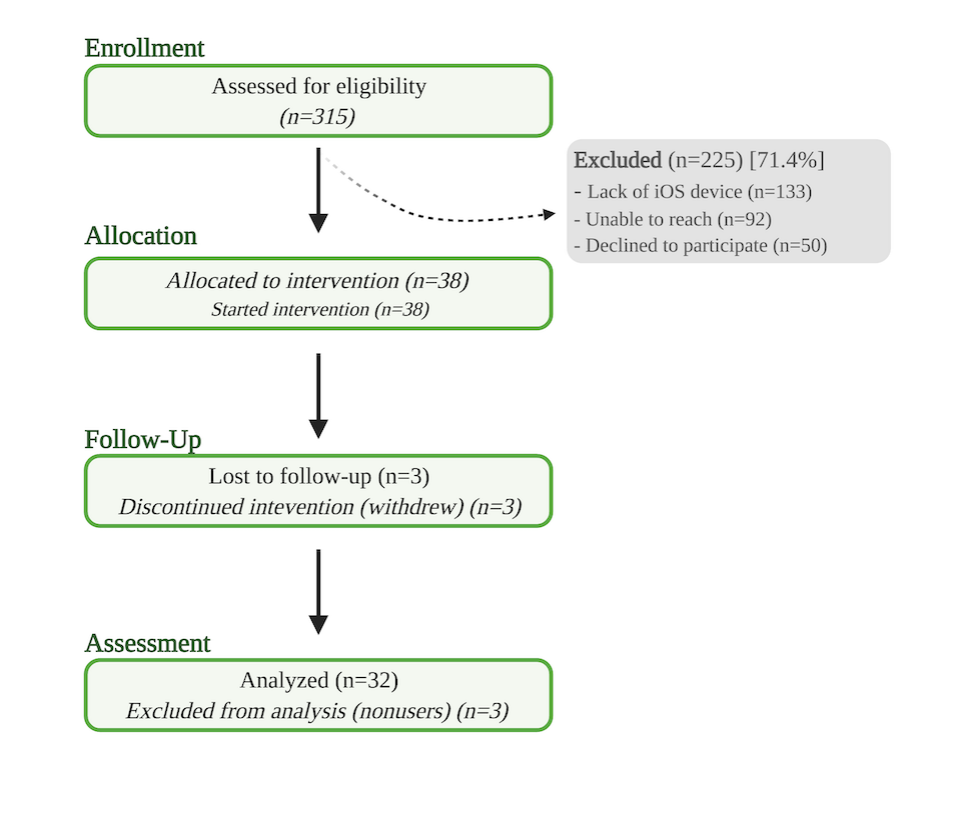
CONSORT flowchart for this study.

A total of 315 patients were screened, of which 90 (28.6%) met the eligibility criteria. Of the 90 patients, 38 (42%) enrolled in the study. The primary reason for ineligibility was the lack of an iPhone device (133/315, 42.2%). The primary reason that eligible patients declined to participate was limited time and disinterest (50/90, 56%). Attrition rate was 16% (6/38) consisting of 3 dropouts and 3 classifieds as nonusers (participants who did not complete at least one PRO questionnaire during the study period) (Figure 3).

### Participant Characteristics

Participants were predominantly males (26/32, 81%), Whites (25/32, 78%), and married (21/32, 66%), reflecting the regional demographics of HNC. The mean age of the participants was 57.8 (SD 12.3) years (range 24-77 years), and the median time from treatment completion to study enrollment was 10.8 months (range 1-23 months). The majority were college educated, including some college (7/32, 22%), bachelor’s degree (9/32, 28%), or higher (9/32, 28%) (Table 1).

**Table 1:** Sociodemographic and clinical characteristics of the participants (N=32).

Characteristics^a,b^		Values, n (%)
**Gender**		
	Female	6 (19)
	Male	26 (81)
**Ethnicity**		
	White	25 (78)
	Black	3 (9)
	Asian	2 (6)
	Other	2 (6)
**Marital status**		
	Single	5 (16)
	Married	21 (66)
	Divorced	5 (16)
	Widow	1 (3)
**Highest level of education**		
	High school/GED	7 (22)
	Some college	7 (22)
	Bachelors or equivalent	9 (28)
	> Bachelor’s	9 (28)
**Type of treatment**		
	Radiation	6 (19)
	Chemotherapy	14 (44)
	Combination	12 (38)
**Self-reported physical activity** **compared to others in their age group**		
	More	6 (19)
	Less	6 (19)
	The same	20 (63)
**Self-reported physical health**		
	Poor/Fair	8 (25)
	Good	14 (44)
	Very Good/Excellent	10 (31)
**Facility**		
	Site 1	26 (81)
	Site 2	6 (19)

^a^Mean (SD) age in years = 57.8 (12.3) years.

^b^Mean (SD) time since last treatment = 10.8 (8.0) months.

### App Use Tracking

Of the 32 participants eligible for analysis, 17 (53%) completed all of the scheduled sessions, 20 (63%) completed 75% or more of the sessions, and 25 (78%) completed at least 50% of the scheduled sessions by the end of the study period. Overall, 73.2% (375/512) of the questionnaires were completed (range 6.25%-100%) with participants opening the app 693 times over the course of 8 weeks.

### Postintervention Survey

#### Usability

At the end of the study, 17 of the 32 participants (53%) reconsented to complete the SUS. The mean SUS score (95% CI) was 71.9 (64.3-79.5), which was an “acceptable” rating based on the standard SUS [28]. Further analysis of the subscales showed that the mean SUS (95% CI) learnability domain was 78.7 (71.2-86.1) and the mean (95% CI) usability domain was 70.2 (61.8-78.7). In the responses to the SUS questionnaire, 88% (15/17) found LogPAL “easy to use,” 94% (16/17) felt that “most people could learn to use LogPAL very quickly,” and 82% (14/17) felt “very confident using the system.”

#### Acceptability

Among the participants, 76% (13/17) agreed that LogPAL was useful, with 59% (10/17) and 71% (12/17) agreeing with the frequency and length of the PROs, respectively. Additionally, 76% (13/17) of the participants agreed that they would recommend LogPAL to other cancer survivors. A total of 5 (29%) of the 17 participants gave additional feedback on their experience using LogPAL. In terms of what they liked, participants used the words “informative,” “helpful,” and “valuable.” One patient stated, “I thought it was definitely helpful, it really let you understand what was going on with your body and it just wasn’t you experiencing these symptoms.” In terms of areas of improvements, participants mentioned the need for the app to be available to patients during or immediately after treatment, an alert tone as a reminder to update, and the ability to provide additional comments on changes. As one patient mentioned, the app “...does not allow for comments when an answer changes due to new variable in my situation, to explain why I changed my answer from previous weeks.”

### Exploratory Analyses

#### Additional App Features

Engagement with additional app features were reviewed among eligible participants. Findings showed that 32 (100%) used *View My* Progress, 27 (84%) used the *Self-Care Tips,* and 5 (16%) used the *Resource* feature. During the study period, the *View My Progress* feature was clicked 3445 times and the *Self-Care Tips* was clicked 79 times.

#### Relationship Among Covariates

Analysis of mean (SD) adherence scores and participant characteristics showed higher scores among those who self-reported conducting “more” physical activity (16.0 [SD 0.0]) than among those who self-reported “about the same” (12.2 [SD 4.9]) as others their age (*P*=.04). Similarly, the scores were higher among participants who self-reported their physical health as “very good”/“excellent” (14.5 [SD 4.4]) than among those who self-reported their physical health as “fair”/“poor” (7.8 [SD 2.1]) (*P*=.05). Lastly, the mean (SD) scores of the participants at clinic site 1 (13.2 [SD 4.5]) were higher than those of the participants at clinic site 2 (5.8 [SD 5.7]) (*P*=.003).

## Discussion

### Principal Results

To the best of our knowledge, this is the first study to evaluate the feasibility of a patient-facing mobile app that collects ePROs specifically for HNC survivors. Our results indicate that participants considered LogPAL as a feasible approach by meeting the a priori criteria. Our findings highlight the potential that mHealth apps have to improve symptom control and promote self-management of symptoms to improve health outcomes and quality of life. These findings are encouraging now more than ever, as the COVID-19 pandemic has placed greater importance on remote technology-enabled monitoring of high-risk patients through a digital platform. We believe that there is a greater need to develop an approach that allows HNC survivors to feel that their unique symptom needs are met and to have the ability to access straightforward information and valued material that address relevant issues.

### Comparison With Prior Work

With the ubiquity of mobile phones in our society, there is a growing interest in and use of mobiles apps for patients with cancer to self-manage symptoms during treatment and those that persist into survivorship [37]. These apps have the potential to provide individually tailored self-management advice for different participants during their survivorship at home [38]. Furthermore, mHealth apps can increase patient engagement in their own recovery, provide better patient-provider communication, and flag patients at risk for readmission, thereby facilitating potential early interventions. Results from this study demonstrate that participant adherence (17/32, 53%) was congruent with that reported in previous studies that defined usage as over 50% of the participants that actually use an eHealth PRO intervention as intended. Other recent single-arm pilot trials have shown higher adherence rates; 1 mHealth ePRO study among patients with prostate cancer found that 86% (25/29) of the participants satisfactorily completed 60% of the weekly questions over a 3-month period [39]. Another pilot study of 10 patients with gynecological cancers who received palliative chemotherapy showed >70% adherence to daily smartphone surveys >4 days per week [40]. One explanatory factor for this inconsistency is the lack of clarity regarding evaluation methodologies, leading to substantial heterogeneity in the reported outcomes [41]. Recent attempts to meaningfully summarize indicators used in pilot studies have led to the creation of a lexicon of the most commonly used terms to identify effective app components [34,42]; however, a conceptually coherent framework is yet to be adapted. Another possibility to explain the lower engagement is the relationship between usage and descriptive variables such as age, marital status, years of education, and socioeconomic status [43]. Concomitantly, we did not find a significant relationship between ePRO usage among patients with HNC and the common descriptive mediating factors [44]. However, additional variables such as physical health, physical activity, and site of recruitment were not previously investigated. As such, these findings were unexpected and suggest that those who have self-perceived better physical health and those who are more physically active than those of their age may lead to higher engagement. It is important to note that there is strong evidence to show that usage of mobile phones and wearable devices increases physical activity and health, which significantly reduces cancer-related symptoms/side effects, leads to greater quality of life [45,46], as well as good retention rates and adherence. However, further investigation is needed to determine the relationships.

Additionally, clinic site 2 had notably lower retention and adherence rates compared to clinic site 1. Clinic site 2 is located in an ambulatory clinic within an urban hospital with 1 surgeon seeing those patients. In contrast, clinic site 1 is located in an outpatient ambulatory clinic in a suburban hospital campus, with 3 different surgeons seeing patients. The recruitment staff at site 1 were different from those at site 2. Lastly, our results corroborated the need to develop cancer-supportive digital interventions that are interactive and tailored [47]. Integrating relevant information such as *Self Care Tips* as participants progress has been suggested as a way to maintain and enhance patients’ experience of personal relevance [42]. In the future iterations, it might be beneficial to integrate an option for participants to include or supplant symptoms or concerns in PROs to make it more person-facing.

### Limitations

This study is not without limitations. First, the app is currently only available on iPhone operating system (Apple iPhone and iPad) devices, which potentially caused selection bias (during enrollment) for age, ethnicity/race, education, and socioeconomic factors. Second, although the demographic characteristics of our cohort were slightly more heterogenous than those in most cancer pilot studies [48], this study was conducted in a single multi-site health system, thereby limiting its generalizability and not typifying those of the wider population. Third, patients who were enrolled in the study may be healthier than a general HNC follow-up population, thereby potentially biasing the engagement and usage figures to appear higher than they would be in a generalized population. Lastly, more rigorous recruitment protocols are needed to ascertain equitable retention rates. Additional updates and development of an Android version will help reduce these biases.

### Conclusions

This study has provided preliminary evidence to suggest that the LogPAL app is a feasible and acceptable mHealth intervention that collects PROs to improve symptom management and proactively detect serious downstream complications among HNC survivors. The success of this feasibility study presents support for conducting a larger, multisite, randomized clinical trial to assess the efficacy of LogPAL with an active control and more heterogenous sample size.

## Abbreviations

ePRO: electronic patient-reported outcome
HNC: head and neck cancer
mHealth: mobile health
PRO: patient-reported outcome
REDCap: Research Electronic Data Capture
SUS: system usability scale
